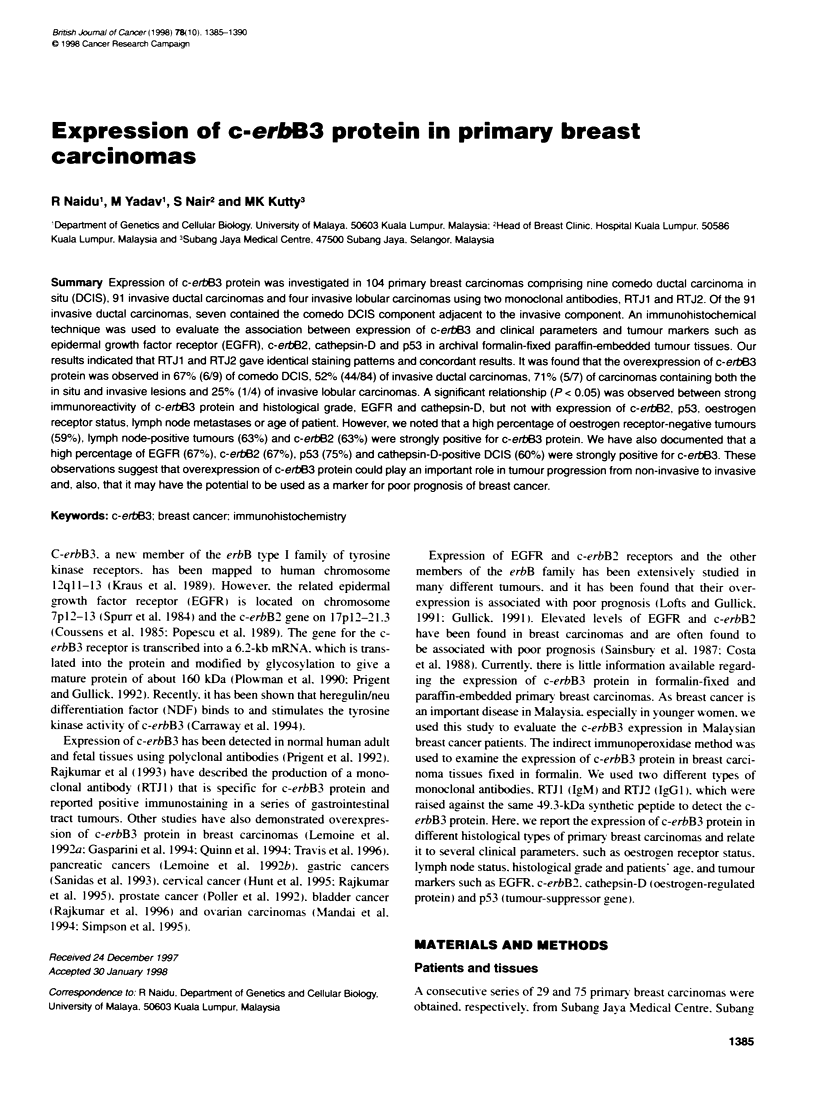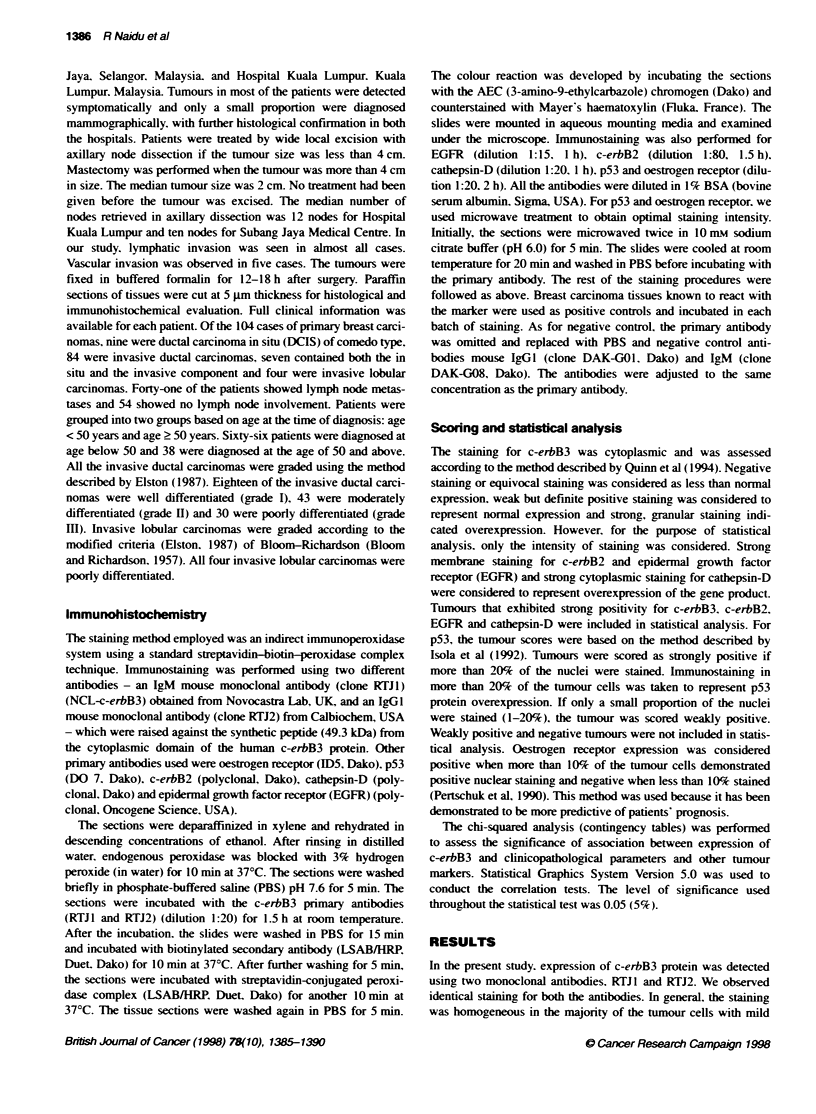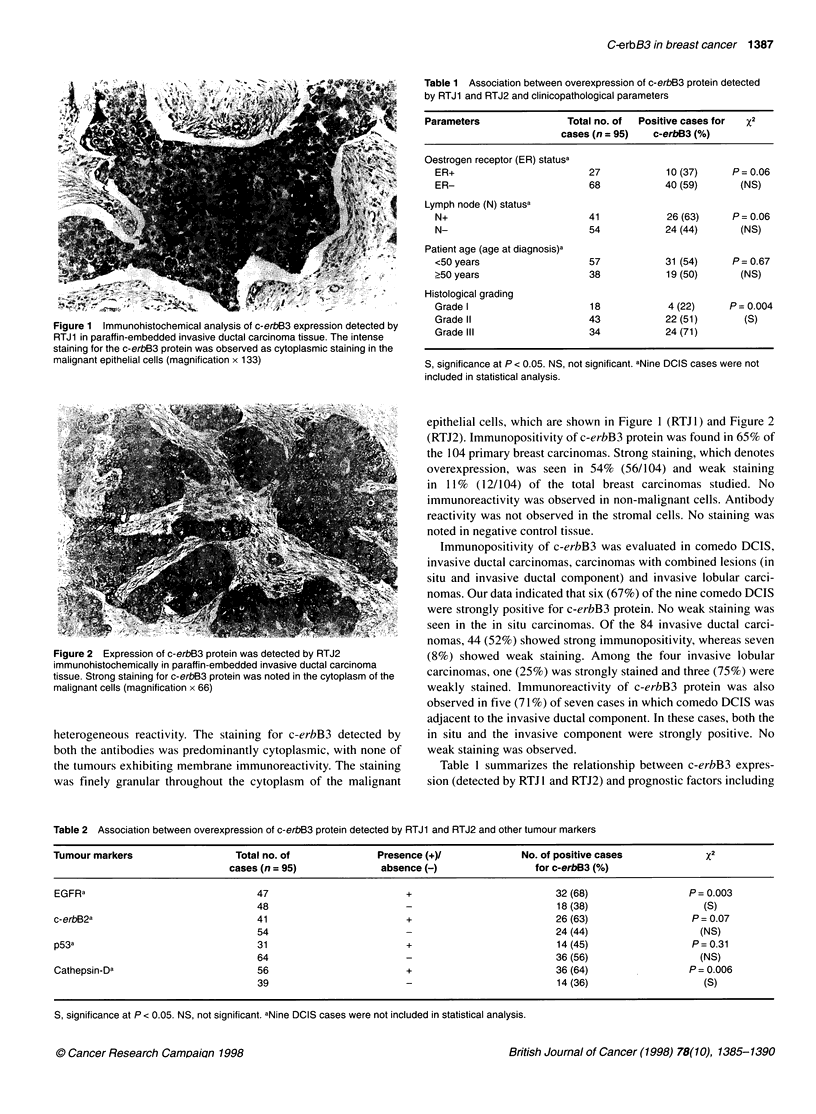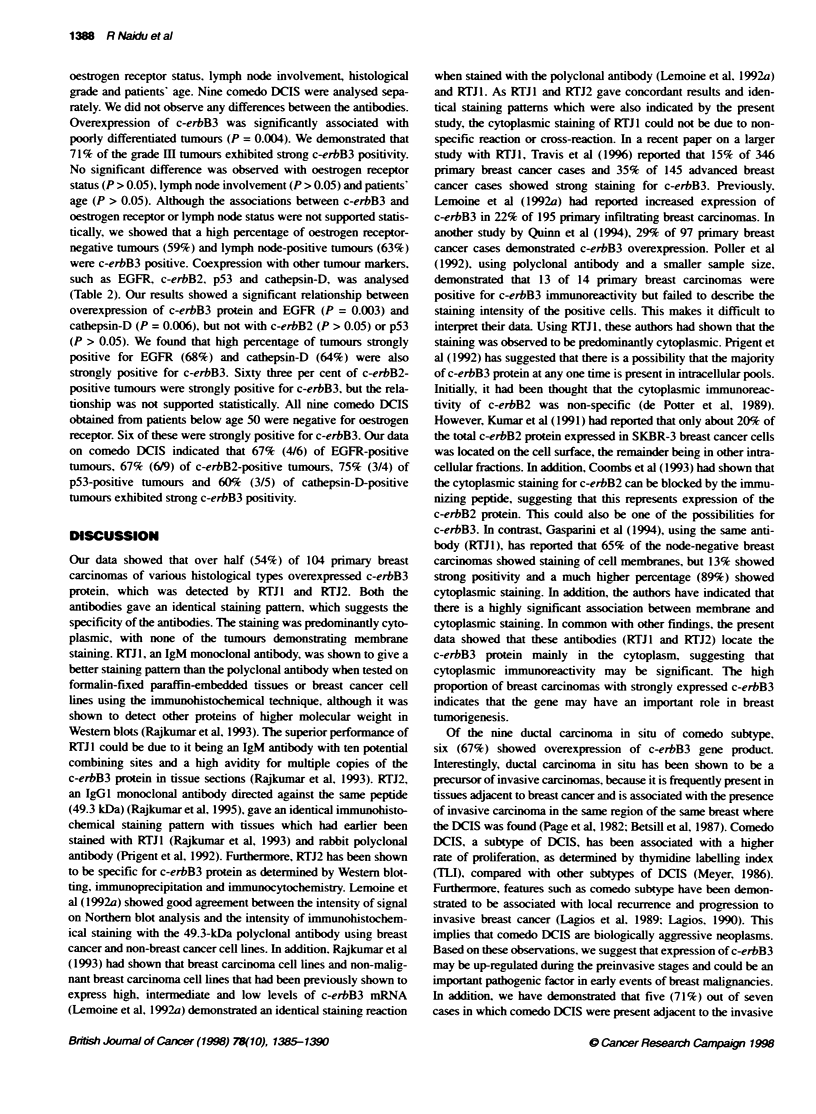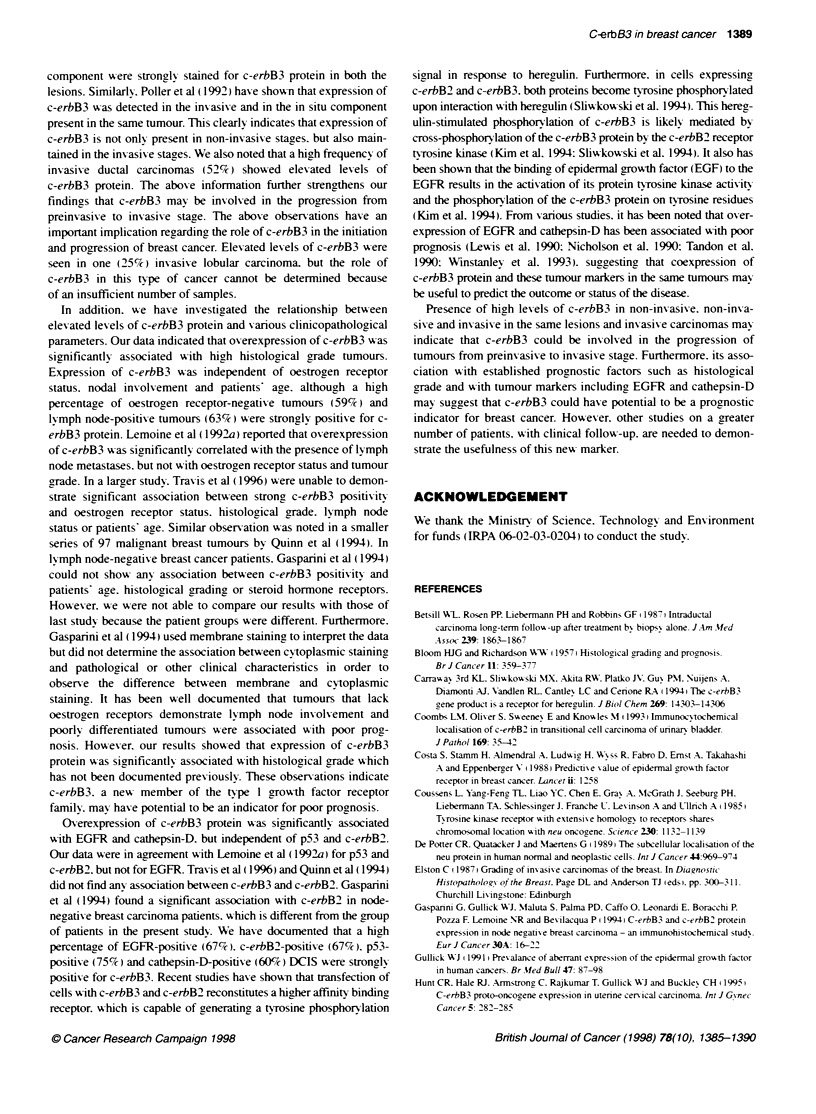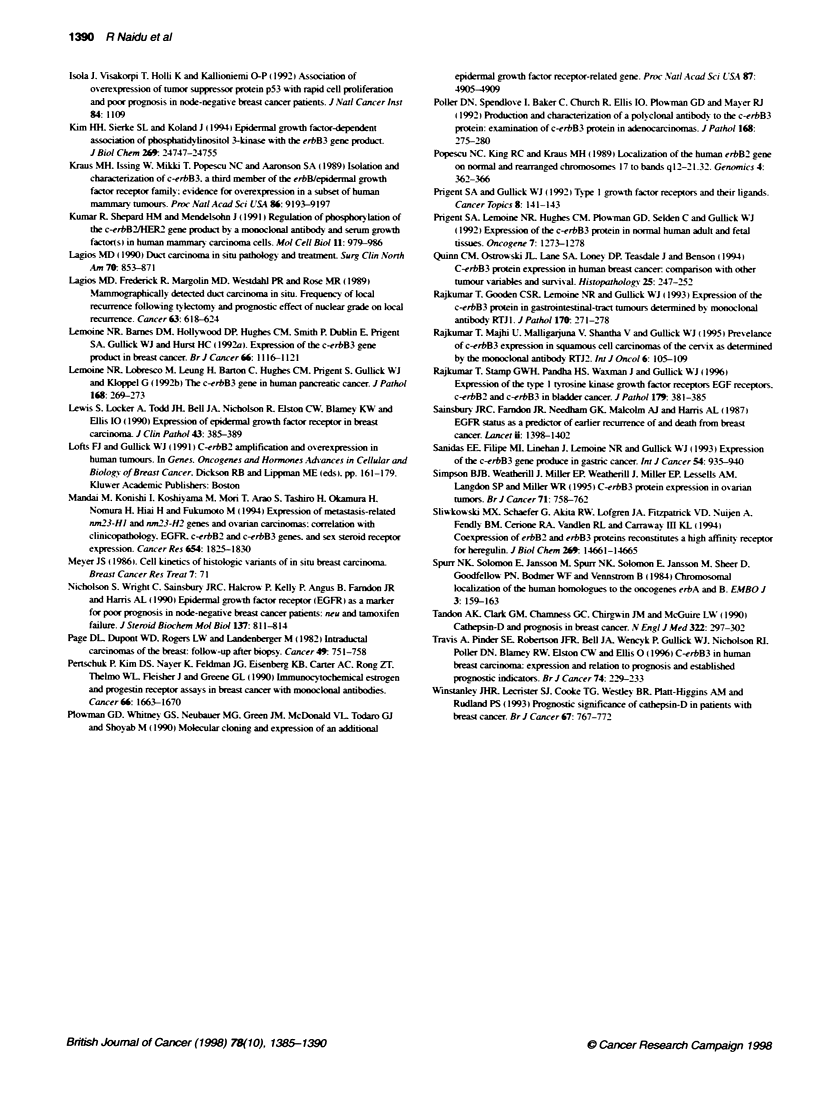# Expression of c-erbB3 protein in primary breast carcinomas.

**DOI:** 10.1038/bjc.1998.689

**Published:** 1998-11

**Authors:** R. Naidu, M. Yadav, S. Nair, M. K. Kutty

**Affiliations:** Department of Genetics and Cellular Biology, University of Malaya, Kuala Lumpur, Malaysia.

## Abstract

**Images:**


British Journal of Cancer (1998) 78(10). 1385–1390
© 1998 Cancer Research Campaign

### Expression of c-*erb*B3 protein in primary breast carcinomas

**R Naidu[1], M Yadav[1], S Nair[2] and MK Kutty[3]**

[1]Department of Genetics and Cellular Biology. University of Malaya. 50603 Kuala Lumpur. Malaysia: [2]Head of Breast Clinic. Hospital Kuala Lumpur. 50586 Kuala Lumpur. Malaysia and [3]Subang Jaya Medical Centre. 47500 Subang Jaya. Selangor. Malaysia

**Summary** Expression of c-*erb*B3 protein was investigated in 104 primary breast carcinomas comprising nine comedo ductal carcinoma in situ (DCIS), 91 invasive ductal carcinomas and four invasive lobular carcinomas using two monoclonal antibodies, RTJ1 and RTJ2. Of the 91 invasive ductal carcinomas, seven contained the comedo DCIS component adjacent to the invasive component. An immunohistochemical technique was used to evaluate the association between expression of c-*erb*B3 and clinical parameters and tumour markers such as epidermal growth factor receptor (EGFR), c-*erb*B2, cathepsin-D and p53 in archival formalin-fixed paraffin-embedded tumour tissues. Our results indicated that RTJ1 and RTJ2 gave identical staining patterns and concordant results. It was found that the overexpression of c-*erb*B3 protein was observed in 67% (6/9) of comedo DCIS, 52% (44/84) of invasive ductal carcinomas, 71% (5/7) of carcinomas containing both the in situ and invasive lesions and 25% (1/4) of invasive lobular carcinomas. A significant relationship ($P < 0.05$) was observed between strong immunoreactivity of c-*erb*B3 protein and histological grade, EGFR and cathepsin-D, but not with expression of c-*erb*B2, p53, oestrogen receptor status, lymph node metastases or age of patient. However, we noted that a high percentage of oestrogen receptor-negative tumours (59%), lymph node-positive tumours (63%) and c-*erb*B2 (63%) were strongly positive for c-*erb*B3 protein. We have also documented that a high percentage of EGFR (67%), c-*erb*B2 (67%), p53 (75%) and cathepsin-D-positive DCIS (60%) were strongly positive for c-*erb*B3. These observations suggest that overexpression of c-*erb*B3 protein could play an important role in tumour progression from non-invasive to invasive and, also, that it may have the potential to be used as a marker for poor prognosis of breast cancer.

**Keywords:** c-*erb*B3; breast cancer; immunohistochemistry

C-*erb*B3. a new member of the *erb*B type I family of tyrosine kinase receptors. has been mapped to human chromosome 12q11–13 (Kraus et al. 1989). However. the related epidermal growth factor receptor (EGFR) is located on chromosome 7p12–13 (Spurr et al. 1984) and the c-*erb*B2 gene on 17p12–21.3 (Coussens et al. 1985: Popescu et al. 1989). The gene for the c-*erb*B3 receptor is transcribed into a 6.2-kb mRNA. which is translated into the protein and modified by glycosylation to give a mature protein of about 160 kDa (Plowman et al. 1990: Prigent and Gullick. 1992). Recently. it has been shown that heregulin/neu differentiation factor (NDF) binds to and stimulates the tyrosine kinase activity of c-*erb*B3 (Carraway et al. 1994).

Expression of c-*erb*B3 has been detected in normal human adult and fetal tissues using polyclonal antibodies (Prigent et al. 1992). Rajkumar et al (1993) have described the production of a monoclonal antibody (RTJ1) that is specific for c-*erb*B3 protein and reported positive immunostaining in a series of gastrointestinal tract tumours. Other studies have also demonstrated overexpression of c-*erb*B3 protein in breast carcinomas (Lemoine et al. 1992*a*: Gasparini et al. 1994: Quinn et al. 1994: Travis et al. 1996). pancreatic cancers (Lemoine et al. 1992*b*). gastric cancers (Sanidas et al. 1993). cervical cancer (Hunt et al. 1995: Rajkumar et al. 1995). prostate cancer (Poller et al. 1992). bladder cancer (Rajkumar et al. 1996) and ovarian carcinomas (Mandai et al. 1994: Simpson et al. 1995).

*Received 24 December 1997*
*Accepted 30 January 1998*

*Correspondence to:* R Naidu. Department of Genetics and Cellular Biology. University of Malaya. 50603 Kuala Lumpur. Malaysia

Expression of EGFR and c-*erb*B2 receptors and the other members of the *erb*B family has been extensively studied in many different tumours. and it has been found that their overexpression is associated with poor prognosis (Lofts and Gullick. 1991: Gullick. 1991). Elevated levels of EGFR and c-*erb*B2 have been found in breast carcinomas and are often found to be associated with poor prognosis (Sainsbury et al. 1987: Costa et al. 1988). Currently. there is little information available regarding the expression of c-*erb*B3 protein in formalin-fixed and paraffin-embedded primary breast carcinomas. As breast cancer is an important disease in Malaysia. especially in younger women. we used this study to evaluate the c-*erb*B3 expression in Malaysian breast cancer patients. The indirect immunoperoxidase method was used to examine the expression of c-*erb*B3 protein in breast carcinoma tissues fixed in formalin. We used two different types of monoclonal antibodies. RTJ1 (IgM) and RTJ2 (IgG1). which were raised against the same 49.3-kDa synthetic peptide to detect the c-*erb*B3 protein. Here. we report the expression of c-*erb*B3 protein in different histological types of primary breast carcinomas and relate it to several clinical parameters. such as oestrogen receptor status. lymph node status. histological grade and patients' age. and tumour markers such as EGFR. c-*erb*B2. cathepsin-D (oestrogen-regulated protein) and p53 (tumour-suppressor gene).

### MATERIALS AND METHODS

### Patients and tissues

A consecutive series of 29 and 75 primary breast carcinomas were obtained. respectively. from Subang Jaya Medical Centre. Subang

Jaya, Selangor, Malaysia, and Hospital Kuala Lumpur, Kuala Lumpur, Malaysia. Tumours in most of the patients were detected symptomatically and only a small proportion were diagnosed mammographically, with further histological confirmation in both the hospitals. Patients were treated by wide local excision with axillary node dissection if the tumour size was less than 4 cm. Mastectomy was performed when the tumour was more than 4 cm in size. The median tumour size was 2 cm. No treatment had been given before the tumour was excised. The median number of nodes retrieved in axillary dissection was 12 nodes for Hospital Kuala Lumpur and ten nodes for Subang Jaya Medical Centre. In our study, lymphatic invasion was seen in almost all cases. Vascular invasion was observed in five cases. The tumours were fixed in buffered formalin for 12–18 h after surgery. Paraffin sections of tissues were cut at 5 μm thickness for histological and immunohistochemical evaluation. Full clinical information was available for each patient. Of the 104 cases of primary breast carcinomas, nine were ductal carcinoma in situ (DCIS) of comedo type, 84 were invasive ductal carcinomas, seven contained both the in situ and the invasive component and four were invasive lobular carcinomas. Forty-one of the patients showed lymph node metastases and 54 showed no lymph node involvement. Patients were grouped into two groups based on age at the time of diagnosis: age < 50 years and age ≥ 50 years. Sixty-six patients were diagnosed at age below 50 and 38 were diagnosed at the age of 50 and above. All the invasive ductal carcinomas were graded using the method described by Elston (1987). Eighteen of the invasive ductal carcinomas were well differentiated (grade I), 43 were moderately differentiated (grade II) and 30 were poorly differentiated (grade III). Invasive lobular carcinomas were graded according to the modified criteria (Elston, 1987) of Bloom–Richardson (Bloom and Richardson, 1957). All four invasive lobular carcinomas were poorly differentiated.

### Immunohistochemistry

The staining method employed was an indirect immunoperoxidase system using a standard streptavidin–biotin–peroxidase complex technique. Immunostaining was performed using two different antibodies – an IgM mouse monoclonal antibody (clone RTJ1) (NCL-c-*erb*B3) obtained from Novocastra Lab, UK, and an IgG1 mouse monoclonal antibody (clone RTJ2) from Calbiochem, USA – which were raised against the synthetic peptide (49.3 kDa) from the cytoplasmic domain of the human c-*erb*B3 protein. Other primary antibodies used were oestrogen receptor (ID5, Dako), p53 (DO 7, Dako), c-*erb*B2 (polyclonal, Dako), cathepsin-D (polyclonal, Dako) and epidermal growth factor receptor (EGFR) (polyclonal, Oncogene Science, USA).

The sections were deparaffinized in xylene and rehydrated in descending concentrations of ethanol. After rinsing in distilled water, endogenous peroxidase was blocked with 3% hydrogen peroxide (in water) for 10 min at 37°C. The sections were washed briefly in phosphate-buffered saline (PBS) pH 7.6 for 5 min. The sections were incubated with the c-*erb*B3 primary antibodies (RTJ1 and RTJ2) (dilution 1:20) for 1.5 h at room temperature. After the incubation, the slides were washed in PBS for 15 min and incubated with biotinylated secondary antibody (LSAB/HRP, Duet, Dako) for 10 min at 37°C. After further washing for 5 min, the sections were incubated with streptavidin-conjugated peroxidase complex (LSAB/HRP, Duet, Dako) for another 10 min at 37°C. The tissue sections were washed again in PBS for 5 min. The colour reaction was developed by incubating the sections with the AEC (3-amino-9-ethylcarbazole) chromogen (Dako) and counterstained with Mayer's haematoxylin (Fluka, France). The slides were mounted in aqueous mounting media and examined under the microscope. Immunostaining was also performed for EGFR (dilution 1:15, 1 h), c-*erb*B2 (dilution 1:80, 1.5 h), cathepsin-D (dilution 1:20, 1 h), p53 and oestrogen receptor (dilution 1:20, 2 h). All the antibodies were diluted in 1% BSA (bovine serum albumin, Sigma, USA). For p53 and oestrogen receptor, we used microwave treatment to obtain optimal staining intensity. Initially, the sections were microwaved twice in 10 mM sodium citrate buffer (pH 6.0) for 5 min. The slides were cooled at room temperature for 20 min and washed in PBS before incubating with the primary antibody. The rest of the staining procedures were followed as above. Breast carcinoma tissues known to react with the marker were used as positive controls and incubated in each batch of staining. As for negative control, the primary antibody was omitted and replaced with PBS and negative control antibodies mouse IgG1 (clone DAK-G01, Dako) and IgM (clone DAK-G08, Dako). The antibodies were adjusted to the same concentration as the primary antibody.

### Scoring and statistical analysis

The staining for c-*erb*B3 was cytoplasmic and was assessed according to the method described by Quinn et al (1994). Negative staining or equivocal staining was considered as less than normal expression, weak but definite positive staining was considered to represent normal expression and strong, granular staining indicated overexpression. However, for the purpose of statistical analysis, only the intensity of staining was considered. Strong membrane staining for c-*erb*B2 and epidermal growth factor receptor (EGFR) and strong cytoplasmic staining for cathepsin-D were considered to represent overexpression of the gene product. Tumours that exhibited strong positivity for c-*erb*B3, c-*erb*B2, EGFR and cathepsin-D were included in statistical analysis. For p53, the tumour scores were based on the method described by Isola et al (1992). Tumours were scored as strongly positive if more than 20% of the nuclei were stained. Immunostaining in more than 20% of the tumour cells was taken to represent p53 protein overexpression. If only a small proportion of the nuclei were stained (1–20%), the tumour was scored weakly positive. Weakly positive and negative tumours were not included in statistical analysis. Oestrogen receptor expression was considered positive when more than 10% of the tumour cells demonstrated positive nuclear staining and negative when less than 10% stained (Pertschuk et al, 1990). This method was used because it has been demonstrated to be more predictive of patients' prognosis.

The chi-squared analysis (contingency tables) was performed to assess the significance of association between expression of c-*erb*B3 and clinicopathological parameters and other tumour markers. Statistical Graphics System Version 5.0 was used to conduct the correlation tests. The level of significance used throughout the statistical test was 0.05 (5%).

### RESULTS

In the present study, expression of c-*erb*B3 protein was detected using two monoclonal antibodies, RTJ1 and RTJ2. We observed identical staining for both the antibodies. In general, the staining was homogeneous in the majority of the tumour cells with mild

© Cancer Research Campaign 1998

**Figure 1** Immunohistochemical analysis of c-*erb*B3 expression detected by RTJ1 in paraffin-embedded invasive ductal carcinoma tissue. The intense staining for the c-*erb*B3 protein was observed as cytoplasmic staining in the malignant epithelial cells (magnification × 133)

**Table 1** Association between overexpression of c-*erb*B3 protein detected by RTJ1 and RTJ2 and clinicopathological parameters

| Parameters | Total no. of cases ($n$ = 95) | Positive cases for c-*erb*B3 (%) | $\chi^2$ |
|---|---|---|---|
| Oestrogen receptor (ER) status[a] | | | |
| ER+ | 27 | 10 (37) | $P$ = 0.06 |
| ER– | 68 | 40 (59) | (NS) |
| Lymph node (N) status[a] | | | |
| N+ | 41 | 26 (63) | $P$ = 0.06 |
| N– | 54 | 24 (44) | (NS) |
| Patient age (age at diagnosis)[a] | | | |
| <50 years | 57 | 31 (54) | $P$ = 0.67 |
| ≥50 years | 38 | 19 (50) | (NS) |
| Histological grading | | | |
| Grade I | 18 | 4 (22) | $P$ = 0.004 |
| Grade II | 43 | 22 (51) | (S) |
| Grade III | 34 | 24 (71) | |

S, significance at $P < 0.05$. NS, not significant. [a]Nine DCIS cases were not included in statistical analysis.

**Figure 2** Expression of c-*erb*B3 protein was detected by RTJ2 immunohistochemically in paraffin-embedded invasive ductal carcinoma tissue. Strong staining for c-*erb*B3 protein was noted in the cytoplasm of the malignant cells (magnification × 66)

heterogeneous reactivity. The staining for c-*erb*B3 detected by both the antibodies was predominantly cytoplasmic, with none of the tumours exhibiting membrane immunoreactivity. The staining was finely granular throughout the cytoplasm of the malignant epithelial cells, which are shown in Figure 1 (RTJ1) and Figure 2 (RTJ2). Immunopositivity of c-*erb*B3 protein was found in 65% of the 104 primary breast carcinomas. Strong staining, which denotes overexpression, was seen in 54% (56/104) and weak staining in 11% (12/104) of the total breast carcinomas studied. No immunoreactivity was observed in non-malignant cells. Antibody reactivity was not observed in the stromal cells. No staining was noted in negative control tissue.

Immunopositivity of c-*erb*B3 was evaluated in comedo DCIS, invasive ductal carcinomas, carcinomas with combined lesions (in situ and invasive ductal component) and invasive lobular carcinomas. Our data indicated that six (67%) of the nine comedo DCIS were strongly positive for c-*erb*B3 protein. No weak staining was seen in the in situ carcinomas. Of the 84 invasive ductal carcinomas, 44 (52%) showed strong immunopositivity, whereas seven (8%) showed weak staining. Among the four invasive lobular carcinomas, one (25%) was strongly stained and three (75%) were weakly stained. Immunoreactivity of c-*erb*B3 protein was also observed in five (71%) of seven cases in which comedo DCIS was adjacent to the invasive ductal component. In these cases, both the in situ and the invasive component were strongly positive. No weak staining was observed.

Table 1 summarizes the relationship between c-*erb*B3 expression (detected by RTJ1 and RTJ2) and prognostic factors including

**Table 2** Association between overexpression of c-*erb*B3 protein detected by RTJ1 and RTJ2 and other tumour markers

| Tumour markers | Total no. of cases ($n$ = 95) | Presence (+)/ absence (–) | No. of positive cases for c-*erb*B3 (%) | $\chi^2$ |
|---|---|---|---|---|
| EGFR[a] | 47 | + | 32 (68) | $P$ = 0.003 |
| | 48 | – | 18 (38) | (S) |
| c-*erb*B2[a] | 41 | + | 26 (63) | $P$ = 0.07 |
| | 54 | – | 24 (44) | (NS) |
| p53[a] | 31 | + | 14 (45) | $P$ = 0.31 |
| | 64 | – | 36 (56) | (NS) |
| Cathepsin-D[a] | 56 | + | 36 (64) | $P$ = 0.006 |
| | 39 | – | 14 (36) | (S) |

S, significance at $P < 0.05$. NS, not significant. [a]Nine DCIS cases were not included in statistical analysis.

© Cancer Research Campaign 1998

oestrogen receptor status, lymph node involvement, histological grade and patients' age. Nine comedo DCIS were analysed separately. We did not observe any differences between the antibodies. Overexpression of c-*erb*B3 was significantly associated with poorly differentiated tumours ($P = 0.004$). We demonstrated that 71% of the grade III tumours exhibited strong c-*erb*B3 positivity. No significant difference was observed with oestrogen receptor status ($P > 0.05$), lymph node involvement ($P > 0.05$) and patients' age ($P > 0.05$). Although the associations between c-*erb*B3 and oestrogen receptor or lymph node status were not supported statistically, we showed that a high percentage of oestrogen receptor-negative tumours (59%) and lymph node-positive tumours (63%) were c-*erb*B3 positive. Coexpression with other tumour markers, such as EGFR, c-*erb*B2, p53 and cathepsin-D, was analysed (Table 2). Our results showed a significant relationship between overexpression of c-*erb*B3 protein and EGFR ($P = 0.003$) and cathepsin-D ($P = 0.006$), but not with c-*erb*B2 ($P > 0.05$) or p53 ($P > 0.05$). We found that high percentage of tumours strongly positive for EGFR (68%) and cathepsin-D (64%) were also strongly positive for c-*erb*B3. Sixty three per cent of c-*erb*B2-positive tumours were strongly positive for c-*erb*B3, but the relationship was not supported statistically. All nine comedo DCIS obtained from patients below age 50 were negative for oestrogen receptor. Six of these were strongly positive for c-*erb*B3. Our data on comedo DCIS indicated that 67% (4/6) of EGFR-positive tumours, 67% (6/9) of c-*erb*B2-positive tumours, 75% (3/4) of p53-positive tumours and 60% (3/5) of cathepsin-D-positive tumours exhibited strong c-*erb*B3 positivity.

### DISCUSSION

Our data showed that over half (54%) of 104 primary breast carcinomas of various histological types overexpressed c-*erb*B3 protein, which was detected by RTJ1 and RTJ2. Both the antibodies gave an identical staining pattern, which suggests the specificity of the antibodies. The staining was predominantly cytoplasmic, with none of the tumours demonstrating membrane staining. RTJ1, an IgM monoclonal antibody, was shown to give a better staining pattern than the polyclonal antibody when tested on formalin-fixed paraffin-embedded tissues or breast cancer cell lines using the immunohistochemical technique, although it was shown to detect other proteins of higher molecular weight in Western blots (Rajkumar et al, 1993). The superior performance of RTJ1 could be due to it being an IgM antibody with ten potential combining sites and a high avidity for multiple copies of the c-*erb*B3 protein in tissue sections (Rajkumar et al, 1993). RTJ2, an IgG1 monoclonal antibody directed against the same peptide (49.3 kDa) (Rajkumar et al, 1995), gave an identical immunohistochemical staining pattern with tissues which had earlier been stained with RTJ1 (Rajkumar et al, 1993) and rabbit polyclonal antibody (Prigent et al, 1992). Furthermore, RTJ2 has been shown to be specific for c-*erb*B3 protein as determined by Western blotting, immunoprecipitation and immunocytochemistry. Lemoine et al (1992*a*) showed good agreement between the intensity of signal on Northern blot analysis and the intensity of immunohistochemical staining with the 49.3-kDa polyclonal antibody using breast cancer and non-breast cancer cell lines. In addition, Rajkumar et al (1993) had shown that breast carcinoma cell lines and non-malignant breast carcinoma cell lines that had been previously shown to express high, intermediate and low levels of c-*erb*B3 mRNA (Lemoine et al, 1992*a*) demonstrated an identical staining reaction when stained with the polyclonal antibody (Lemoine et al, 1992*a*) and RTJ1. As RTJ1 and RTJ2 gave concordant results and identical staining patterns which were also indicated by the present study, the cytoplasmic staining of RTJ1 could not be due to non-specific reaction or cross-reaction. In a recent paper on a larger study with RTJ1, Travis et al (1996) reported that 15% of 346 primary breast cancer cases and 35% of 145 advanced breast cancer cases showed strong staining for c-*erb*B3. Previously, Lemoine et al (1992*a*) had reported increased expression of c-*erb*B3 in 22% of 195 primary infiltrating breast carcinomas. In another study by Quinn et al (1994), 29% of 97 primary breast cancer cases demonstrated c-*erb*B3 overexpression. Poller et al (1992), using polyclonal antibody and a smaller sample size, demonstrated that 13 of 14 primary breast carcinomas were positive for c-*erb*B3 immunoreactivity but failed to describe the staining intensity of the positive cells. This makes it difficult to interpret their data. Using RTJ1, these authors had shown that the staining was observed to be predominantly cytoplasmic. Prigent et al (1992) has suggested that there is a possibility that the majority of c-*erb*B3 protein at any one time is present in intracellular pools. Initially, it had been thought that the cytoplasmic immunoreactivity of c-*erb*B2 was non-specific (de Potter et al, 1989). However, Kumar et al (1991) had reported that only about 20% of the total c-*erb*B2 protein expressed in SKBR-3 breast cancer cells was located on the cell surface, the remainder being in other intracellular fractions. In addition, Coombs et al (1993) had shown that the cytoplasmic staining for c-*erb*B2 can be blocked by the immunizing peptide, suggesting that this represents expression of the c-*erb*B2 protein. This could also be one of the possibilities for c-*erb*B3. In contrast, Gasparini et al (1994), using the same antibody (RTJ1), has reported that 65% of the node-negative breast carcinomas showed staining of cell membranes, but 13% showed strong positivity and a much higher percentage (89%) showed cytoplasmic staining. In addition, the authors have indicated that there is a highly significant association between membrane and cytoplasmic staining. In common with other findings, the present data showed that these antibodies (RTJ1 and RTJ2) locate the c-*erb*B3 protein mainly in the cytoplasm, suggesting that cytoplasmic immunoreactivity may be significant. The high proportion of breast carcinomas with strongly expressed c-*erb*B3 indicates that the gene may have an important role in breast tumorigenesis.

Of the nine ductal carcinoma in situ of comedo subtype, six (67%) showed overexpression of c-*erb*B3 gene product. Interestingly, ductal carcinoma in situ has been shown to be a precursor of invasive carcinomas, because it is frequently present in tissues adjacent to breast cancer and is associated with the presence of invasive carcinoma in the same region of the same breast where the DCIS was found (Page et al, 1982; Betsill et al, 1987). Comedo DCIS, a subtype of DCIS, has been associated with a higher rate of proliferation, as determined by thymidine labelling index (TLI), compared with other subtypes of DCIS (Meyer, 1986). Furthermore, features such as comedo subtype have been demonstrated to be associated with local recurrence and progression to invasive breast cancer (Lagios et al, 1989; Lagios, 1990). This implies that comedo DCIS are biologically aggressive neoplasms. Based on these observations, we suggest that expression of c-*erb*B3 may be up-regulated during the preinvasive stages and could be an important pathogenic factor in early events of breast malignancies. In addition, we have demonstrated that five (71%) out of seven cases in which comedo DCIS were present adjacent to the invasive

© Cancer Research Campaign 1998

component were strongly stained for c-*erb*B3 protein in both the lesions. Similarly, Poller et al (1992) have shown that expression of c-*erb*B3 was detected in the invasive and in the in situ component present in the same tumour. This clearly indicates that expression of c-*erb*B3 is not only present in non-invasive stages, but also maintained in the invasive stages. We also noted that a high frequency of invasive ductal carcinomas (52%) showed elevated levels of c-*erb*B3 protein. The above information further strengthens our findings that c-*erb*B3 may be involved in the progression from preinvasive to invasive stage. The above observations have an important implication regarding the role of c-*erb*B3 in the initiation and progression of breast cancer. Elevated levels of c-*erb*B3 were seen in one (25%) invasive lobular carcinoma, but the role of c-*erb*B3 in this type of cancer cannot be determined because of an insufficient number of samples.

In addition, we have investigated the relationship between elevated levels of c-*erb*B3 protein and various clinicopathological parameters. Our data indicated that overexpression of c-*erb*B3 was significantly associated with high histological grade tumours. Expression of c-*erb*B3 was independent of oestrogen receptor status, nodal involvement and patients' age, although a high percentage of oestrogen receptor-negative tumours (59%) and lymph node-positive tumours (63%) were strongly positive for c-*erb*B3 protein. Lemoine et al (1992*a*) reported that overexpression of c-*erb*B3 was significantly correlated with the presence of lymph node metastases, but not with oestrogen receptor status and tumour grade. In a larger study, Travis et al (1996) were unable to demonstrate significant association between strong c-*erb*B3 positivity and oestrogen receptor status, histological grade, lymph node status or patients' age. Similar observation was noted in a smaller series of 97 malignant breast tumours by Quinn et al (1994). In lymph node-negative breast cancer patients, Gasparini et al (1994) could not show any association between c-*erb*B3 positivity and patients' age, histological grading or steroid hormone receptors. However, we were not able to compare our results with those of last study because the patient groups were different. Furthermore, Gasparini et al (1994) used membrane staining to interpret the data but did not determine the association between cytoplasmic staining and pathological or other clinical characteristics in order to observe the difference between membrane and cytoplasmic staining. It has been well documented that tumours that lack oestrogen receptors demonstrate lymph node involvement and poorly differentiated tumours were associated with poor prognosis. However, our results showed that expression of c-*erb*B3 protein was significantly associated with histological grade which has not been documented previously. These observations indicate c-*erb*B3, a new member of the type 1 growth factor receptor family, may have potential to be an indicator for poor prognosis.

Overexpression of c-*erb*B3 protein was significantly associated with EGFR and cathepsin-D, but independent of p53 and c-*erb*B2. Our data were in agreement with Lemoine et al (1992*a*) for p53 and c-*erb*B2, but not for EGFR. Travis et al (1996) and Quinn et al (1994) did not find any association between c-*erb*B3 and c-*erb*B2. Gasparini et al (1994) found a significant association with c-*erb*B2 in node-negative breast carcinoma patients, which is different from the group of patients in the present study. We have documented that a high percentage of EGFR-positive (67%), c-*erb*B2-positive (67%), p53-positive (75%) and cathepsin-D-positive (60%) DCIS were strongly positive for c-*erb*B3. Recent studies have shown that transfection of cells with c-*erb*B3 and c-*erb*B2 reconstitutes a higher affinity binding receptor, which is capable of generating a tyrosine phosphorylation signal in response to heregulin. Furthermore, in cells expressing c-*erb*B2 and c-*erb*B3, both proteins become tyrosine phosphorylated upon interaction with heregulin (Sliwkowski et al, 1994). This heregulin-stimulated phosphorylation of c-*erb*B3 is likely mediated by cross-phosphorylation of the c-*erb*B3 protein by the c-*erb*B2 receptor tyrosine kinase (Kim et al, 1994; Sliwkowski et al, 1994). It also has been shown that the binding of epidermal growth factor (EGF) to the EGFR results in the activation of its protein tyrosine kinase activity and the phosphorylation of the c-*erb*B3 protein on tyrosine residues (Kim et al, 1994). From various studies, it has been noted that overexpression of EGFR and cathepsin-D has been associated with poor prognosis (Lewis et al, 1990; Nicholson et al, 1990; Tandon et al, 1990; Winstanley et al, 1993), suggesting that coexpression of c-*erb*B3 protein and these tumour markers in the same tumours may be useful to predict the outcome or status of the disease.

Presence of high levels of c-*erb*B3 in non-invasive, non-invasive and invasive in the same lesions and invasive carcinomas may indicate that c-*erb*B3 could be involved in the progression of tumours from preinvasive to invasive stage. Furthermore, its association with established prognostic factors such as histological grade and with tumour markers including EGFR and cathepsin-D may suggest that c-*erb*B3 could have potential to be a prognostic indicator for breast cancer. However, other studies on a greater number of patients, with clinical follow-up, are needed to demonstrate the usefulness of this new marker.

### ACKNOWLEDGEMENT

We thank the Ministry of Science, Technology and Environment for funds (IRPA 06-02-03-0204) to conduct the study.

### REFERENCES

Betsill WL, Rosen PP, Liebermann PH and Robbins GF (1987) Intraductal carcinoma long-term follow-up after treatment by biopsy alone. *J Am Med Assoc* **239**: 1863–1867

Bloom HJG and Richardson WW (1957) Histological grading and prognosis. *Br J Cancer* **11**: 359–377

Carraway 3rd KL, Sliwkowski MX, Akita RW, Platko JV, Guy PM, Nuijens A, Diamonti AJ, Vandlen RL, Cantley LC and Cerione RA (1994) The c-*erb*B3 gene product is a receptor for heregulin. *J Biol Chem* **269**: 14303–14306

Coombs LM, Oliver S, Sweeney E and Knowles M (1993) Immunocytochemical localisation of c-*erb*B2 in transitional cell carcinoma of urinary bladder. *J Pathol* **169**: 35–42

Costa S, Stamm H, Almendral A, Ludwig H, Wyss R, Fabro D, Ernst A, Takahashi A and Eppenberger V (1988) Predictive value of epidermal growth factor receptor in breast cancer. *Lancet* **ii**: 1258

Coussens L, Yang-Feng TL, Liao YC, Chen E, Gray A, McGrath J, Seeburg PH, Liebermann TA, Schlessinger J, Franche U, Levinson A and Ullrich A (1985) Tyrosine kinase receptor with extensive homology to receptors shares chromosomal location with *neu* oncogene. *Science* **230**: 1132–1139

De Potter CR, Quatacker J and Maertens G (1989) The subcellular localisation of the neu protein in human normal and neoplastic cells. *Int J Cancer* **44**:969–974

Elston C (1987) Grading of invasive carcinomas of the breast. In *Diagnostic Histopathology of the Breast*. Page DL and Anderson TJ (eds), pp. 300–311. Churchill Livingstone: Edinburgh

Gasparini G, Gullick WJ, Maluta S, Palma PD, Caffo O, Leonardi E, Boracchi P, Pozza F, Lemoine NR and Bevilacqua P (1994) C-*erb*B3 and c-*erb*B2 protein expression in node negative breast carcinoma – an immunohistochemical study. *Eur J Cancer* **30A**: 16–22

Gullick WJ (1991) Prevalance of aberrant expression of the epidermal growth factor in human cancers. *Br Med Bull* **47**: 87–98

Hunt CR, Hale RJ, Armstrong C, Rajkumar T, Gullick WJ and Buckley CH (1995) C-*erb*B3 proto-oncogene expression in uterine cervical carcinoma. *Int J Gynec Cancer* **5**: 282–285

Isola J, Visakorpi T, Holli K and Kallioniemi O-P (1992) Association of overexpression of tumor suppressor protein p53 with rapid cell proliferation and poor prognosis in node-negative breast cancer patients. *J Natl Cancer Inst* **84**: 1109

Kim HH, Sierke SL and Koland J (1994) Epidermal growth factor-dependent association of phosphatidylinositol 3-kinase with the *erb*B3 gene product. *J Biol Chem* **269**: 24747–24755

Kraus MH, Issing W, Mikki T, Popescu NC and Aaronson SA (1989) Isolation and characterization of c-*erb*B3, a third member of the *erb*B/epidermal growth factor receptor family: evidence for overexpression in a subset of human mammary tumours. *Proc Natl Acad Sci USA* **86**: 9193–9197

Kumar R, Shepard HM and Mendelsohn J (1991) Regulation of phosphorylation of the c-*erb*B2/HER2 gene product by a monoclonal antibody and serum growth factor(s) in human mammary carcinoma cells. *Mol Cell Biol* **11**: 979–986

Lagios MD (1990) Duct carcinoma in situ pathology and treatment. *Surg Clin North Am* **70**: 853–871

Lagios MD, Frederick R, Margolin MD, Westdahl PR and Rose MR (1989) Mammographically detected duct carcinoma in situ. Frequency of local recurrence following tylectomy and prognostic effect of nuclear grade on local recurrence. *Cancer* **63**: 618–624

Lemoine NR, Barnes DM, Hollywood DP, Hughes CM, Smith P, Dublin E, Prigent SA, Gullick WJ and Hurst HC (1992*a*). Expression of the c-*erb*B3 gene product in breast cancer. *Br J Cancer* **66**: 1116–1121

Lemoine NR, Lobresco M, Leung H, Barton C, Hughes CM, Prigent S, Gullick WJ and Kloppel G (1992b) The c-*erb*B3 gene in human pancreatic cancer. *J Pathol* **168**: 269–273

Lewis S, Locker A, Todd JH, Bell JA, Nicholson R, Elston CW, Blamey KW and Ellis IO (1990) Expression of epidermal growth factor receptor in breast carcinoma. *J Clin Pathol* **43**: 385–389

Lofts FJ and Gullick WJ (1991) C-*erb*B2 amplification and overexpression in human tumours. In *Genes, Oncogenes and Hormones Advances in Cellular and Biology of Breast Cancer*, Dickson RB and Lippman ME (eds), pp. 161–179. Kluwer Academic Publishers: Boston

Mandai M, Konishi I, Koshiyama M, Mori T, Arao S, Tashiro H, Okamura H, Nomura H, Hiai H and Fukumoto M (1994) Expression of metastasis-related *nm23-H1* and *nm23-H2* genes and ovarian carcinomas: correlation with clinicopathology, EGFR, c-*erb*B2 and c-*erb*B3 genes, and sex steroid receptor expression. *Cancer Res* **654**: 1825–1830

Meyer JS (1986). Cell kinetics of histologic variants of in situ breast carcinoma. *Breast Cancer Res Treat* **7**: 71

Nicholson S, Wright C, Sainsbury JRC, Halcrow P, Kelly P, Angus B, Farndon JR and Harris AL (1990) Epidermal growth factor receptor (EGFR) as a marker for poor prognosis in node-negative breast cancer patients: *neu* and tamoxifen failure. *J Steroid Biochem Mol Biol* **137**: 811–814

Page DL, Dupont WD, Rogers LW and Landenberger M (1982) Intraductal carcinomas of the breast: follow-up after biopsy. *Cancer* **49**: 751–758

Pertschuk P, Kim DS, Nayer K, Feldman JG, Eisenberg KB, Carter AC, Rong ZT, Thelmo WL, Fleisher J and Greene GL (1990) Immunocytochemical estrogen and progestin receptor assays in breast cancer with monoclonal antibodies. *Cancer* **66**: 1663–1670

Plowman GD, Whitney GS, Neubauer MG, Green JM, McDonald VL, Todaro GJ and Shoyab M (1990) Molecular cloning and expression of an additional epidermal growth factor receptor-related gene. *Proc Natl Acad Sci USA* **87**: 4905–4909

Poller DN, Spendlove I, Baker C, Church R, Ellis IO, Plowman GD and Mayer RJ (1992) Production and characterization of a polyclonal antibody to the c-*erb*B3 protein: examination of c-*erb*B3 protein in adenocarcinomas. *J Pathol* **168**: 275–280

Popescu NC, King RC and Kraus MH (1989) Localization of the human *erb*B2 gene on normal and rearranged chromosomes 17 to bands q12–21.32. *Genomics* **4**: 362–366

Prigent SA and Gullick WJ (1992) Type 1 growth factor receptors and their ligands. *Cancer Topics* **8**: 141–143

Prigent SA, Lemoine NR, Hughes CM, Plowman GD, Selden C and Gullick WJ (1992) Expression of the c-*erb*B3 protein in normal human adult and fetal tissues. *Oncogene* **7**: 1273–1278

Quinn CM, Ostrowski JL, Lane SA, Loney DP, Teasdale J and Benson (1994) C-*erb*B3 protein expression in human breast cancer: comparison with other tumour variables and survival. *Histopathology* **25**: 247–252

Rajkumar T, Gooden CSR, Lemoine NR and Gullick WJ (1993) Expression of the c-*erb*B3 protein in gastrointestinal-tract tumours determined by monoclonal antibody RTJ1. *J Pathol* **170**: 271–278

Rajkumar T, Majhi U, Malligarjuna V, Shantha V and Gullick WJ (1995) Prevelance of c-*erb*B3 expression in squamous cell carcinomas of the cervix as determined by the monoclonal antibody RTJ2. *Int J Oncol* **6**: 105–109

Rajkumar T, Stamp GWH, Pandha HS, Waxman J and Gullick WJ (1996) Expression of the type 1 tyrosine kinase growth factor receptors EGF receptors, c-*erb*B2 and c-*erb*B3 in bladder cancer. *J Pathol* **179**: 381–385

Sainsbury JRC, Farndon JR, Needham GK, Malcolm AJ and Harris AL (1987) EGFR status as a predictor of earlier recurrence of and death from breast cancer. *Lancet* **ii**: 1398–1402

Sanidas EE, Filipe MI, Linehan J, Lemoine NR and Gullick WJ (1993) Expression of the c-*erb*B3 gene produce in gastric cancer. *Int J Cancer* **54**: 935–940

Simpson BJB, Weatherill J, Miller EP, Weatherill J, Miller EP, Lessells AM, Langdon SP and Miller WR (1995) C-*erb*B3 protein expression in ovarian tumors. *Br J Cancer* **71**: 758–762

Sliwkowski MX, Schaefer G, Akita RW, Lofgren JA, Fitzpatrick VD, Nuijen A, Fendly BM, Cerione RA, Vandlen RL and Carraway III KL (1994) Coexpression of *erb*B2 and *erb*B3 proteins reconstitutes a high affinity receptor for heregulin. *J Biol Chem* **269**: 14661–14665

Spurr NK, Solomon E, Jansson M, Spurr NK, Solomon E, Jansson M, Sheer D, Goodfellow PN, Bodmer WF and Vennstrom B (1984) Chromosomal localization of the human homologues to the oncogenes *erb*A and B. *EMBO J* **3**: 159–163

Tandon AK, Clark GM, Chamness GC, Chirgwin JM and McGuire LW (1990) Cathepsin-D and prognosis in breast cancer. *N Engl J Med* **322**: 297–302

Travis A, Pinder SE, Robertson JFR, Bell JA, Wencyk P, Gullick WJ, Nicholson RI, Poller DN, Blamey RW, Elston CW and Ellis O (1996) C-*erb*B3 in human breast carcinoma: expression and relation to prognosis and established prognostic indicators. *Br J Cancer* **74**: 229–233

Winstanley JHR, Lecrister SJ, Cooke TG, Westley BR, Platt-Higgins AM and Rudland PS (1993) Prognostic significance of cathepsin-D in patients with breast cancer. *Br J Cancer* **67**: 767–772

*© Cancer Research Campaign 1998*